# Functional and morphological effects of laser-induced ocular hypertension in retinas of adult albino Swiss mice

**Published:** 2009-12-05

**Authors:** Manuel Salinas-Navarro, Luis Alarcón-Martínez, Francisco Javier Valiente-Soriano, Arturo Ortín-Martínez, Manuel Jiménez-López, Marcelino Avilés-Trigueros, María Paz Villegas-Pérez, Pedro de la Villa, Manuel Vidal-Sanz

**Affiliations:** 1 Departamento de Oftalmología, Facultad de Medicina, Universidad de Murcia, E-30100 Murcia, Spain; 2 Departamento de Fisiología, Facultad de Medicina, Universidad de Alcalá, E-28871 Alcalá de Henares, Spain

## Abstract

**Purpose:**

To investigate the effects of laser photocoagulation (LP)-induced ocular hypertension (OHT) on the survival and retrograde axonal transport of retinal ganglion cells (RGC), as well as on the function of retinal layers.

**Methods:**

Adult albino Swiss mice (35–45 g) received laser photocoagulation of limbal and episcleral veins in the left eye. Mice were sacrificed at 8, 17, 35, and 63 days. Intraocular pressure (IOP) in both eyes was measured with a Tono-Lab before LP and at various days after LP. Flash electroretinogram (ERG) scotopic threshold response (STR) and a- and b-wave amplitudes were recorded before LP and at  various  times after LP. RGCs were labeled with 10% hydroxystilbamidine methanesulfonate (OHSt) applied to both superior colliculi before sacrifice and in some mice, with dextran tetramethylrhodamine (DTMR) applied to the ocular stump of the intraorbitally transected optic nerve. Retinas were immunostained for RT97 or Brn3a. Retinas were prepared as whole-mounts and photographed under a fluorescence microscope. Labeled RGCs were counted using image analysis software, and an isodensity contour plot was generated for each retina.

**Results:**

IOP increased to twice its basal values by 24 h and was maintained until day 5, after which IOP gradually declined to reach basal values by 1 wk. Similar IOP increases were observed in all groups. The mean total number of OHSt^+^ RGCs was 13,428±6,295 (n=12), 10,456±14,301 (n=13), 12,622±14,174 (n=21), and 10,451±13,949 (n=13) for groups I, II, III, and IV, respectively; these values represented 28%, 23%, 26%, and 22% of the values found in their contralateral fellow retinas. The mean total population of Brn3a^+^ RGCs was 24,343±5,739 (n=12) and 10,219±8,887 (n=9), respectively, for groups I and III; these values represented 49% and 20%, respectively, of the values found in their fellow eyes. OHT retinas showed an absence of OHSt^+^ and DTMR^+^ RGCs in both focal wedge-shaped and diffuse regions of the retina. By 1 wk, there was a discrepancy between the total number of surviving OHSt^+^ RGCs and Brn3a^+^ RGCs, suggesting that a large proportion of RGCs had impaired retrograde axonal transport. In the retinal areas lacking backlabeled RGCs, neurofibrillar staining revealed aberrant expression of RT97 within axons and RGC bodies characteristic of axotomy. Elevated IOP induced significant reductions in the registered ERG waves, including positive STR, a- and b-waves, that were observed by 24 h and remained throughout the period of study for the three groups analyzed.

**Conclusions:**

LP of the perilimbal and episcleral veins resulted in OHT leading to a lack of retrograde axonal transport in approximately 75% of the original RGC population. This lack did not progress further between 8 and 63 days, and it was both focal (in sectors with the apex located in the optic disc) and diffuse within the retina. In addition, severe amplitude diminutions of the STR and a- and b-waves of the ERG appeared as early as 24 h after lasering and did not recover throughout the period of study, indicating that increased IOP results in severe damage to the innermost, inner nuclear, and outer nuclear layers of the retina.

## Introduction

The mammalian retina is a layered structure [1] that processes and sends to the brain the information obtained from light stimulation in the visual field. This is done with increasing complexity in each of the three main neuronal layers: the outer nuclear layer, where the cell bodies of the rod and cone photoreceptors lie; the intermediate or inner nuclear layer, where the cell bodies of the bipolar, horizontal, interplexiform, and amacrine interneurons reside; and the innermost retinal layer, also known as the retinal ganglion cell layer, where displaced amacrine interneurons and retinal ganglion cells (RGCs), the sole output neurons of the retina, reside [2]. A common disease that affects many of these retinal neurons is glaucoma. Glaucomatous optic neuropathy is a human disease that characteristically affects the RGC population but may also affect other retinal neuronal populations [3], including the inner [4] and outer nuclear layers of the retina [5,6]; it provokes optic disc changes and leads to visual field losses that may progress to complete blindness. Effects on non-RGC neuronal populations in the retina have been shown in several studies that investigated the thickness and functional status of the inner and outer retinal layers both morphologically and with electroretinogram (ERG) recordings [7,8]. Indeed, major wave components of the ERG have been shown to be severely affected in humans with glaucoma [9,10] or ocular hypertension (OHT) [11] as well as in experimental glaucoma [12]. Experimental models of elevated intraocular pressure (IOP) [13-17] have shown important alterations of several ERG components, including scotopic threshold response (STR), a-wave, and b-wave, which are associated with RGCs, photoreceptors, and bipolar cells, respectively.

Even though not all glaucoma visual field loss may be correlated with IOP [18] and lowering IOP does not completely halt progression of the disease [19], elevation of IOP remains one of the most important risk factors associated with the progression of glaucomatous optic neuropathy in humans [20-22]. Up-to-date therapeutic intervention against glaucoma progression is based mainly on medical or surgical treatments to diminish IOP in an attempt to control or slow the progression of the disease and its damaging effects [23,24]. Thus, it is of interest to characterize the effects of elevated IOP on retinal neurons.

In the present work, we have used one of the available experimental models of OHT-induced retinal damage [24,25] to study the mechanisms by which elevated IOP causes retinal damage in adult albino mice. The experimental model we have chosen is based on laser photocoagulation (LP) of the perilimbal and episcleral veins [26], a method originally described to induce OHT in monkeys [27] and later modified for albino rats [26,28-31] and pigmented mice [32,33]. These models do not fully resemble human glaucoma, but they are suitable to improve understanding of the pathophysiology involved in OHT-induced retinal damage [25], as well as to test neuroprotective strategies and substances [25,34,35]. Injured RGCs are known to undergo early functional deficits [17,36-38], including alterations in their axoplasmic flow properties [26,39-43], in several metabolic functions such as diminutions of their *Thy-1* mRNA levels [36,44-46], or induction of phagocytic activity in microglia [47,48], and in the regulation of a substantial number of genes [49,50] including the downregulation of Brn3a [51] shortly before cell death. Transgenic mice are used in increasing numbers as experimental models for various neurodegenerative diseases [25], including the line of DBA/2 mice used as glaucomatous optic neuropathy models [3,52-57]; this, with their availability, their well characterized genome, and the relatively low cost of producing diverse genetic manipulations [58], underscores the need to characterize the effects of ocular hypertension in wild mice, as well. This has been done for pigmented mice [17,32,59-61]. However, there is as yet no information regarding the results of IOP in adult albino mice. Thus, we have here extended our previous observations in the adult albino rat [26] and further characterized the effects of increased IOP in the retinal neuronal population of adult albino mice. Specifically, we have investigated the fate of the RGC population using various neuronal tracers and molecular markers and studied the functional effects in the retinal layers by recording full-field ERGs.

Our studies show, for the first time in adult albino Swiss mice, that lasering of the limbal tissues results in elevated IOP, which in turn induces several sequential degenerative events in the RGC layer that result in degeneration of the RGC population, with a time lapse between the first alteration in the axoplasmic flow and the delayed degeneration of the intraocular axons and their parent cell bodies; this underlines the opportunity for neuroprotective strategies to be applied within a long interval before actual RGC death and is consistent with our recent studies in adult albino rats [26]. Moreover, we show permanent significant diminution of the scotopic threshold response that is associated with the loss of the great majority of RGCs following elevated IOP; this indicates the value of noninvasive functional ERG measurements to document RGC loss in adult rodents. Finally, we show permanent significant diminution of the major components of the ERG, the a-wave, and the b-wave, all of which imply severe alterations of the inner and outer nuclear layers of the retina. Short accounts of this work have been published in abstract form [62,63].

## Methods

### Animals and anesthetics

Experiments were performed on adult male albino Swiss mice (35–45 g), obtained from the breeding colony of the University of Murcia (Murcia, Spain). Mice were housed in temperature- and light-controlled rooms with a 12 h:12 h light-dark cycle and had food and water ad libitum. Light intensity within the cages ranged from 9 to 24 lx. Animal care guidelines comparable those published by the Institute for Laboratory Animal Research (Guide for the Care and Use of Laboratory Animals) and the USA Public Health Service (Public Health Service Policy on Humane Care and Use of Laboratory Animals) were followed. In addition, animal manipulations followed institutional guidelines, European Union regulations for the use of animals in research, and the ARVO statement for the use of animals in ophthalmic and vision research.

All surgical manipulations were performed under general anesthesia induced with an intraperitoneal injection of a mixture of ketamine (75 mg/kg, Ketolar®, Parke-Davies, S.L., Barcelona, Spain) and xylazine (10 mg/kg, Rompún®, Bayer, S.A., Barcelona, Spain). During recovery from anesthesia, rats were placed in their cages, and an ointment containing tobramycin (Tobrex®; Alcon Cusí S.A., Barcelona, Spain) was applied on the cornea to prevent corneal desiccation. Additional measures were taken to minimize discomfort and pain after surgery. Animals were sacrificed with an intraperitoneal injection of an overdose of pentobarbital (Dolethal Vetoquinol®, Especialidades Veterinarias, S.A., Alcobendas, Madrid, Spain).

### Induction of ocular hypertension

To induce ocular hypertension, the left eyes of anesthetized mice were treated during a single session with a series of diode laser (532 nm, Quantel Medical, Clermont-Ferrand, France) burns. The laser beam was delivered directly, without any lenses, aimed at the limbal and episcleral veins. The spot size, duration, and power were 50–100 µm, 0.5 s, and 0.3 W, respectively. Four groups of 12 (group I), 13 (group II), 21 (group III), and 13 (group IV) mice were prepared, each receiving approximately 72 spots in a single session.

### Measurement of intraocular pressure, experimental groups, and survival intervals

To assess IOP in the treated mice, we obtained accurate and reliable readings using the rebound tonometer (Tono-Lab®; Tiolat, OY, Helsinki, Finland) [64,65]. Both eyes of each animal were measured under anesthesia before laser treatment and 1, 2, 3, 4, 5, 6, 7, 8, 14, 21, 28, 42, or 56 days after treatment. At each time point, 36 consecutive readings were performed for each eye, and the results were averaged. To avoid fluctuations due to the circadian rhythm of the mice [33] or to the elevated IOP itself [66], we tested IOP always around the same time, preferentially in the morning and immediately after deep anesthesia. Moroever, because general anesthesia lowers IOP in rats, we measured both the IOP-treated eye and the contralateral intact fellow eye in all cases. Mice were divided into four groups that were sacrificed and analyzed 8 (group I, n=12); 17 (group II, n=13), 35 (group III, n=21), or 63 (group IV, n=13) days after lasering.

### Electroretinography recordings

One of the purposes of this study was to evaluate the effects of OHT on the function of the retina. To this end, we analyzed animals from groups II–IV that were sacrificed at 17, 35, and 63 days, respectively. Animals were dark-adapted for 8 h before ERG recordings, and they were manipulated under dim red light (λ>600 nm). Mice were anesthetized, their IOP was measured as above, and bilateral pupil mydriasis was induced by applying in both eyes a topical drop of 1% tropicamide (Colircusi tropicamida 1%®; Alcon-Cusí, S.A., El Masnou, Barcelona, Spain). The light-stimulation device consisted of a Ganzfeld dome, which ensures homogeneous illumination throughout the retina, with multiple reflections of the light generated by light-emitting diodes (LEDs), providing a wide range of light intensities. For high-intensity illuminations, a single LED placed close (1 mm) to the eye was used. The recording system was composed of Burian-Allen bipolar electrodes (Hansen Labs, Coralville, IA) with a corneal contact shape; a drop of 2% methylcellulose (Methocel 2%®; Novartis Laboratories CIBA Vision, Annonay, France) was placed between the eye and the electrode to maximize conductivity of the generated response. The reference electrode was placed in the mouth and the ground electrode in the tail. Electrical signals generated in the retina were amplified (x1000) and filtered (band pass from 1 Hz to 1000 Hz) using a commercial amplifier (Digitimer Ltd., Letchworth Garden City, UK). The recorded signals were digitized (Power Lab; ADInstruments Pty. Ltd., Chalgrove, UK) and displayed on a PC computer. Bilateral ERG recording was performed simultaneously on both eyes. Light stimuli were calibrated before each experiment, and the calibration protocol ensured the same recording parameters for both eyes. Scotopic ERG responses were recorded by stimulating the retina with light intensities between 10^−6^ and 10^−4^ cd·s·m^−2^ for the scotopic threshold response (STR), between 10^−4^ and 10^−2^ cd·s·m^−2^ for the rod response, and between 10^−2^ and 10^2^ cd·s·m^−2^ for the mixed (rod and cone) response. After five minutes of light adaptation (50 cd·m^−2^ background light), the photopic ERG responses were recorded at a light intensity of 10^2^ cd·s·m^−2^. For each light intensity, a series of ERG responses was averaged, and the interval between light flashes was adjusted to allow response recovery. At the end of each session, the animals were treated with topical tobramycin (Tobrex®; Alcon-Cusí, S.A., El Masnou, Barcelona, Spain) in both eyes. The recordings were analyzed using the normalization criteria established for the ISCEV for the measures of amplitude and implicit time for the various waves that were studied.

The STR was analyzed for each stimuli; positive scotopic threshold response (pSTR) was measured from the baseline to the “hill” of the positive deflection, ~110 ms from the flash onset. The a-wave was measured from the baseline to the first valley, ~10 ms from the flash onset. The b-wave amplitude was measured from the bottom of the a-wave valley to the top of the hill of the positive deflection; the time point of the measurement varied depending on the light intensity used. Implicit time was measured from the presentation of the stimulus to the top of the b-wave. Data from laser-treated and untreated eyes were compared; ERG wave amplitudes and implicit times were calculated for each animal group, II–IV. The results were analyzed with SigmaStat® 3.1 for Windows® (Systat Software Inc., Richmond, CA). Descriptive statistics were calculated and *t*-test was used in all studied groups for the comparison between percent response both pre- and post-lasering. We performed the same tests before surgery to demonstrate similar functionality in both eyes before the lesions. One-way ANOVA was applied for the comparison between different animal groups, as an attempt to estimate a possible interrelation between the progressions of response along studied times. The statistic significance was placed in a p<0.05 for all tests and the statistic was always of two tails.

### Retrograde labeling of retinal ganglion cells with hydroxystilbamidine methanesulfonate

To identify RGCs that retain their axoplasmic transport after lasering, hydroxystilbamidine methanesulfonate (OHSt) (Molecular Probes, Leiden, The Netherlands), a small molecule (472.53 kDa Mw) with similar fluorescent and tracer properties to Fluorogold® [67], was applied to both superior colliculi (SCi) one week before animal sacrifice following previously described methods that are standard in our laboratory [51,68]. In brief, after exposing the midbrain, a small pledget of gelatin sponge (Espongostan Film, Ferrosan A/S, Denmark) soaked in saline containing 10% OHSt in 0.9% NaCl and 10% dimethyl sulphoxide (DMSO) was applied over the entire surface of both SCi [68-71]. Previous studies in control mice indicate that OHSt application to both SCi, which are the main retinorecipient target regions in the brain, results in the labeling of 48,733 RGCs one week later; these represent 98.5% of the RGC population in albino Swiss mice [69].

### Retrograde labeling of retinal ganglion cells with dextran tetramethylrhodamine

To identify RGCs surviving retinal lasering with a competent axon at the level of the optic nerve (ON) head, the fluorescence tracer dextran tetramethylrhodamine (DTMR; 3,000 MW; Molecular Probes, Inc. Eugene, OR) was applied to the ocular stump of the intraorbitally transected left optic nerve 5 days after OHSt application and 2 days before sacrifice in groups II, III, and IV, following already described methods that are standard in our Laboratory [26,43,69,72-74]. In brief, small crystals of DMTR were applied to the ocular stump of the left ON, which had been intraorbitally sectioned approximately 0.5 mm from the eye. Care was taken not to damage the retinal vessels that run in the inferior aspect of the dural sheaths [75,76]. DTMR diffuses through the axon toward the cell soma at a rate of 2 mm/h without requiring active transport [77] producing an intense labeling [28,69]. In these animals, DTMR would label the surviving retinofugal RGC population with a competent axon at the level of the ON head, whereas OHSt would identify RGCs that retain their capability to transport the compound from the SCi back to their cell somas in the retina.

### Animal processing

Mice were deeply anesthetized and perfused transcardially through the ascending aorta with saline and 4% paraformaldehyde in 0.1 M phosphate buffer (PB; 1:1.087 sodium phosphate monobasic and sodium phosphate dibasic at pH 7.2–7.4). The orientation of each eye was carefully maintained with a suture placed on the superior pole immediately after deep anesthesia and before perfusion fixation. Moreover, upon dissection of the eye, the insertion of the rectus muscle and the nasal caruncle were used as additional landmarks. Both retinas were dissected and prepared as flattened whole-mounts by making four radial cuts (the deepest in the superior pole); post-fixed for an additional hour; rinsed in 0.1 M PB; mounted vitreal side up on subbed slides; and covered with antifading mounting media containing 50% glycerol and 0.04% p-phenylenediamine in 0.1 M sodium carbonate buffer (pH 9).

### Immunocytochemical studies

Twelve animals from group I and nine animals from group III (sacrificed 8 and 35 days after lasering, respectively) were further processed for Brn3a immunocytochemistry in whole-mounts following previously described methods [51]. Brn3a is part of the Brn3 family of POU-domain transcription factors, recently shown to be a reliable marker to quantify naïve and axotomized RGCs in adult albino rats [51]. Axotomized RGCs are known to undergo downregulation of the Brn3a gene [49,50] shortly before they die. Moreover, two to three days after optic nerve injury, there is an important decrease in the expression of the protein (as observed by western blotting and immunohistofluorescence) [51]; thus, an indirect way to examine the physiologic functional properties of RGCs is to examine their Brn3a expression. In brief, immediately after retinal dissection, retinas were permeabilized in PBS 0.1 M (pH 7.2–7.4) 0.5% Triton® X-100 by freezing them overnight at −70 °C, rinsed in new PBS 0.5% Triton®X-100, and incubated the following day at 4 °C with goat anti-Brn3a (C-20) antibody (Santa Cruz Biotechnologies Heidelberg, Germany) diluted 1:100 in blocking buffer (PBS, 2% BSA (BAS), 2% Triton®X-100). Then, retinas were washed three times in PBS and incubated 2 h at room temperature (RT) with a secondary antibody, Alexa Fluor-568 donkey antigoat IgG (H^+^L) (Molecular Probes, Invitrogen, Barcelona, Spain), diluted 1:500 in blocking buffer. Finally, retinas were thoroughly washed in PBS, mounted vitreal side up on subbed slides, and covered with antifading mounting media.

The intraretinal course of RGC axons was examined in retinal whole mounts with the monoclonal antibody RT97 (Hybridoma Bank, University of Iowa, Iowa City, IA) in both retinas of animals from groups I–IV. The RT97 antibody developed by John Wood [78] was obtained from the Developmental Studies Hybridoma Bank developed under the auspices of the NICHD and maintained by the University of Iowa, Department of Biologic Sciences, Iowa City, IA. This antibody was raised in mouse against Wistar rat neurofilaments and recognizes in western blots the phosphorylated heaviest subunit (200 kDa) of the neurofilament triplet [78-80]. In our laboratory, we have used this commercial antibody extensively, because, in our experience, RT97 labels RGC axons intensely and the horizontal cell plexus faintly in the rodent whole-mount retina [74,81-86]. Moreover, it is a good marker for axotomized RGCs and their axons [26,74,81-84]. Immunodetection was performed using protocols that are standard in our laboratory [26,71,74,82,83,85,86]. In brief, whole-mount dissected retinas were incubated 1 h at RT in blocking buffer (Triton®X-100 2% and 2.5% BSA in PBS) followed by overnight incubation at 4 °C with the primary antibody diluted 1:1,000 in the same blocking buffer. The following day, retinas were washed in PBS (3×15 min at RT), and secondary detection was performed by 2 h incubation at RT with goat antimouse FITC antibody (F-4018; Sigma-Aldrich, St. Louis, MO) diluted 1:50 in blocking buffer. After that, retinas were washed in PBS, mounted vitreal side up on gelatin-coated slides, with antifading mounting media containing 50% glycerol and 0,04% p-phenylenediamine in 0.1 M sodium carbonate buffer (pH 9).

We performed several negative controls for immunohistochemistry by incubating the retinas without the primary antibodies, and we did not obtain specific signals.

### Retinal analysis

Retinas were examined and photographed under a fluorescence microscope (Axioscop 2 Plus; Zeiss Mikroskopie, Jena, Germany) equipped with an ultraviolet (BP 365/12, LP 397) filter that allows the observation of the white-gold OHSt fluorescence, a rhodamine (BP 546/12, LP 590) filter that allows the observation of the orange-red DTMR fluorescence of the Alexa Fluor-568 donkey antigoat IgG (H^+^L) antibody, and a fluorescein (BP 450/490, LP 515–565) filter that allows the observation of the fluorescein conjugated antibodies. The microscope was also equipped with a digital high-resolution camera (ProgResTM C10; Jenoptik, Jena, Germany) and a computer-driven motorized stage (ProScanTM® H128 Series; Prior Scientific Instruments, Cambridge, UK), connected to an image analysis computer program (Image-Pro Plus 5.1 for Windows (IPP); Media Cybernetics, Silver Spring, MD) with a microscope controller module (Scope-Pro 5.0 for Windows; Media Cybernetics, Silver Spring, MD).

Retinal whole-mount reconstructions were obtained as recently described in detail [68,69]. In brief, retinal multiframe acquisitions were photographed in a raster scan pattern in which frames were captured side-by-side with no gap or overlap between them with a 20X objective (Plan-Neofluar, 20/0.50; Zeiss Mikroskopie, Jena, Germany). Single frames were focused manually before the capture of each image, which was then fed into the image analysis program. A scan area was defined to cover the whole retina, consisting of a matrix of m frames in columns and n frames in rows, where the total number of frames in the scan area is indicated by frames in columns times frames in rows (m×n). Usually, 140 images, each measuring 0.2161 mm^2^ at a resolution of 300 dots per inch, were taken for each mouse retina. The images of each retina were saved in a folder as a set of 24-bit color image pictures, and these images were later combined automatically into a single tiled high-resolution composite image of the whole retina using IPP. Reconstructed images were further processed using image-editing software (Adobe® Photoshop® CS ver. 8.0.1; Adobe Systems Inc., San Jose, CA) when needed to produce printouts.

### Retinal ganglion cell counts and isodensity maps

All images taken from the retinas were processed by specific macros written in IPP that apply a sequence of filters and transformations to each image of the stack in turn before finally counting the resulting cells and transferring the data to a spreadsheet for analysis. These subroutines were recently described in detail [68,69].

The topology of RGCs was analyzed with isodensity maps. Cell densities were calculated and represented as a filled contour plot graph following previously described methods [68,69]. In brief, every captured frame was divided into an equal number of 36 rectangular areas of interest (AOI) for OHSt labeling and 25 AOI for Brn3a labeling. These AOI were automatically counted, and data was exported and saved to a spreadsheet computer program (Microsoft Office Excel 2003; Microsoft Corporation, Redmond, WA). Finally, the data were represented as a filled contour plot using graphing software (SigmaPlot 9.0 for Windows; Systat Software, Inc., Richmond, CA) that constructs pseudocolored isodensity maps in a scale of 45 different steps (each of 125) ranging from 0 to 5,625 cells/mm^2^. This upper limit was chosen on the basis of earlier studies that showed mean highest densities around this value [69].

The RGCs expressing aberrant RT97 staining of their soma were counted in a masked fashion in 5 experimental and 5 naïve retinas from group II, sacrificed 17 days after lasering. This survival interval was chosen because it has been shown in rats that the largest number of RT97 immunopositive (RT97^+^) RGCs occurs between two and three weeks after injury, either by axotomy [74] or by elevated IOP [26]. Digitized retinal whole-mount reconstructions were examined frame by frame at high magnification, and every RT97^+^ RGC was marked with a colored dot to identify either faint or strong RT97 immunoreactivity with the aid of Photoshop® software. A subroutine developed with the IPP image analysis program counted the number of dots (RT97^+^ RGCs) and their topological distribution within each analyzed retina [26,74]. The retinal distribution of RT97^+^ RGCs and OHSt^+^ RGCS within the same retina was examined by comparing their respective topological maps.

### Statistical analysis

In quantitative morphological studies, data are presented as mean±standard deviation (SD) while in quantitative electrophysiological studies, data are represented as mean±standard error of the mean (SEM). Statistical analysis of the differences between groups of retinas or groups of animals was conducted with non-parametric ANOVA tests using Satistix® V1.0 for Windows® 95 (Analytical Software, Tallahassee, FL) software; the Kruskal–Wallis test was used to compare more than two groups, and the Mann–Whitney test was used when comparing two groups only. Differences were considered significant when p<0.05.

## Results

### Laser-induced intraocular pressure values

**Figure 1:**
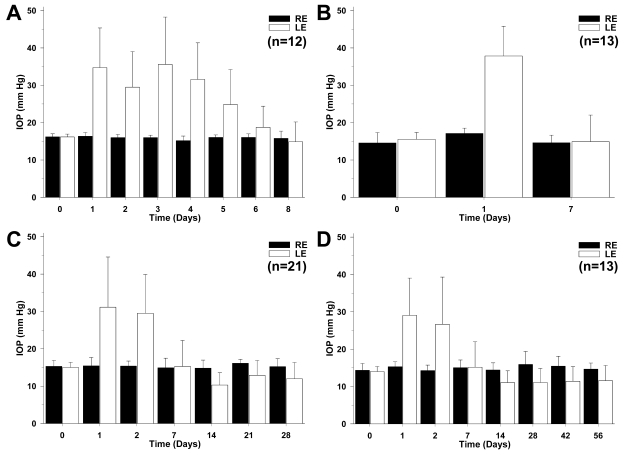
Measurements of intraocular pressure. **A**-**D**: Histograms show mean (±SD) intraocular pressures (IOP) for the right eye (RE) and left eye (LE) in groups I (**A**), II (**B**), III (**C**), and IV (**D**) of mice sacrificed 8, 17, 35, and 63 days, respectively, after laser photocoagulation of the limbal and episcleral veins of the LE. Each received an average of 72 laser-burning spots in a single session. IOP values in group I (**A**) rose and peaked by day 1 post-treatment, were maintained until day 5, and then gradually returned to basal preoperative levels by the end of a week. IOP values in groups II (**B**), III (**C**), and IV (**D**) followed a similar pattern of pressure variations, indicating that elevated IOP was not maintained beyond 1 week.

There was some variability among maximum IOP values in the lasered eyes within individuals and within groups processed at different survival intervals, but the results were generally consistent. Individual IOP measurements in the groups sacrificed at 8, 17, 35, and 63 days after laser treatment are shown in detail in individual histograms for each group (group I; Figure 1A, group II; Figure 1B, group III; Figure 1C, and group IV; Figure 1D). Overall, our data show a similar time course elevation of the IOP for all groups of mice. The statistical analysis showed that within animal groups, IOP values were comparable in the lasered eyes at 24 h (Kruskal–Wallis test, p=0.0891) and 48 h (Kruskal–Wallis test, p=0.9024) after lasering. In general, an important increase of the IOP was already evident and peaked at 24 h after lasering (Kruskal–Wallis test, p<0.0001); this was maintained for four days and returned gradually after the fifth day to the basal value, so that by one week after lasering, the IOP values in animals of different groups were comparable for both eyes (Kruskal–Wallis test, p=0.8191; Figure 1).

### Electroretinography responses

#### ERGs in control albino mice

**Figure 2:**
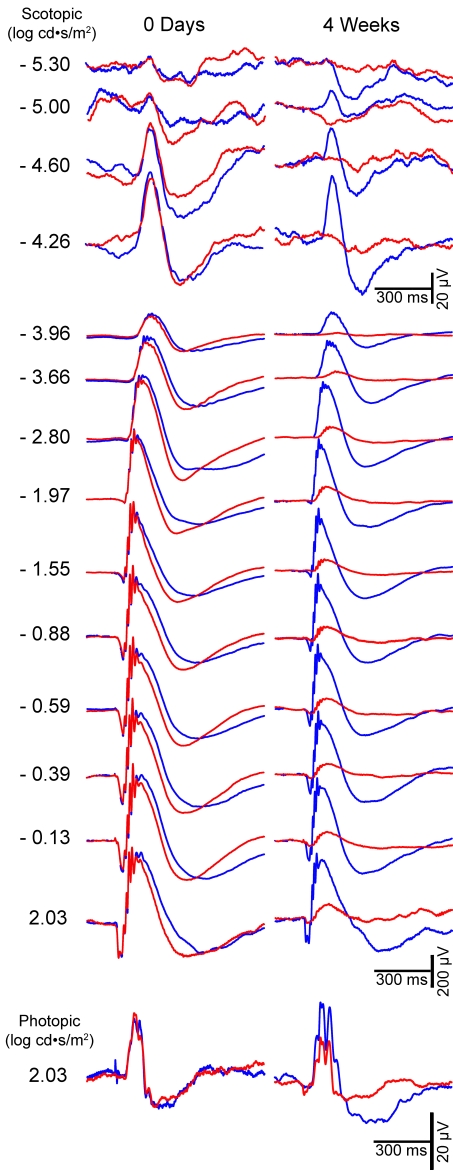
Examples of ERG traces recorded before and four weeks after laser photocoagulation in one representative animal. Electroretinogram recordings in response to flash stimuli of increasing intensity for the untreated right eye (blue traces) and for the lasered left eye (red traces) from one animal in group III. The intensity of the flash stimulus is indicated to the left of each of the recording traces. No difference was observed between the electroretinogram amplitudes of the left and right eyes before laser treatment (0 days), while a significant reduction in all components is clearly visible in the treated eye at four weeks post-treatment (4 weeks), both in scotopic and photopic light conditions.

To study the effect of IOP elevation on the ERG waves in albino mice, simultaneous ERG recordings were performed on the right and left eyes of each animal before surgery. Before surgery, no significant differences were observed between the left and right eyes in any of the animals in the STR, a-wave, and b-wave ERG amplitudes or in the implicit times for the b-waves. Figure 2 shows a representative example of ERG traces recorded in both eyes before surgery in response to flash stimuli of increasing intensity. The STRs were elicited by weak light stimuli (−5.4 to −4.02 log cd·s·m^−2^). The STR amplitude increased exponentially with the intensity of the light stimuli. No ERG a-wave was observed for light intensities below −2.80 log cd·s·m^−2^. The b-wave elicited by light intensities beginning at −3.96 cd·s·m^−2^ also increased exponentially, reaching its maximum for 2.03 cd·s·m^−2^. The photopic ERG response was elicited by a light intensity of 2.03 log cd·s·m^−2^ after 5 min of light adaptation (Figure 2).

#### Electroretinography in experimental albino mice after elevated intraocular pressure

**Figure 3:**
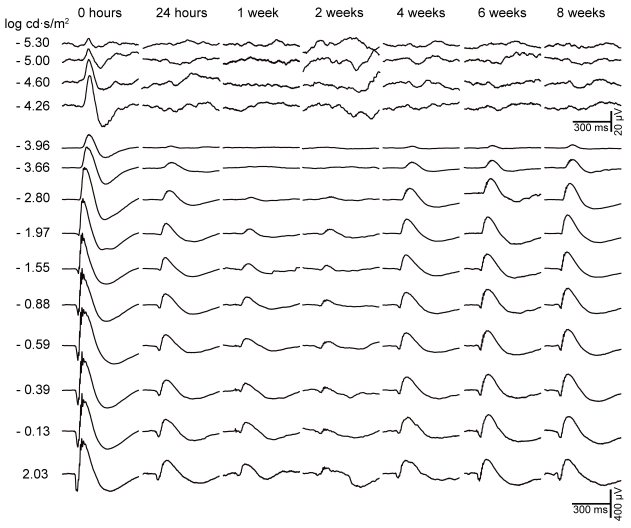
Electroretinogram traces from a laser-treated eye in a representative adult albino Swiss mouse. Electroretinogram recordings in response to flash stimuli of increasing intensity for the from a representative animal in group IV registered before laser treatment (0 h) and 24 h, 1 week, 4 weeks, 6 weeks and 8 weeks later. We found a significant reduction in the scotopic threshold response, a-wave, and b-wave amplitudes by 24 h after lasering, amounting to approximately 81%, 64%, and 54% of their basal values, respectively. The changes observed in the electroretinogram never fully recovered for survival times of up to 8 weeks after lasering, indicating that these are permanent.

**Figure 4:**
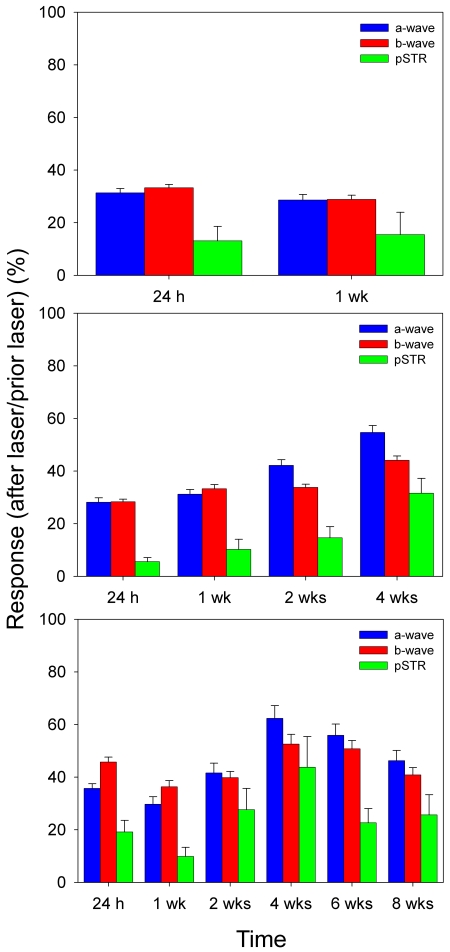
Histograms illustrating evolving changes in the electroretinograms. Average values (mean±SEM) of the positive scotopic threshold response (pSTR) and a- and b-waves are presented as percentages of their basal values in three different experimental groups that were analyzed at increasing survival intervals and sacrificed at 17 (**A**), 35 (**B**), or 63 (**D**) days after lasering. At 24 h after laser treatment, a large reduction can be seen in the amplitudes of all the registered waves when compared to their basal levels. These reductions are maintained over time and do not recover significantly even for the animal group examined at 8 weeks after lasering (**C**), suggesting that laser treatment induces a permanent functional impairment within the innermost, inner and outer retina.

ERG recordings were performed simultaneously for right untreated and left lasered eyes in albino Swiss mice at increasing survival intervals after lasering. Recordings from representative mice in groups III (sacrificed at 35 days) and IV (sacrificed at 63 days) are shown in Figure 2 and Figure 3, respectively. In addition, data are shown as percentages (mean±SEM) of reduction in wave amplitudes in operated eyes with respect to baseline values obtained for the same eyes before surgery (Figure 4). These figures illustrate that reductions in pSTR after lasering are maintained over time. Moreover, the changes observed in the a- and b-wave amplitudes of the scotopic and mixed ERG were obvious at short intervals (7 days and 4 weeks) and were maintained for long periods after lasering (8 weeks). A reduction in the b-wave amplitude of the photopic response was also observed after eye lasering (see photopic response in Figure 2).

### Group II

Mice from this group (n=13) showed pSTR reductions of approximately 87% and 83%, respectively, 1 and 7 days after lasering when compared to their baseline (preoperative values; *t*-test, p<0.001). Scotopic and mixed ERG recorded in these mice 1 and 7 days after lasering showed reduced a-wave amplitudes of 68% and 67% and reduced b-wave amplitudes of 72% and 71%, respectively (*t*-test, p<0.001; Figure 4A).

### Group III

ERG traces from this group (n=18) were recorded before laser treatment and 1, 7, 14, and 28 days after treatment. When compared to their baseline (preoperative values), there were significant (*t*-test, p<0.001) reductions in pSTR of approximately 94%, 83%, 85%, and 60%, respectively, when analyzed at 1, 8, 14, and 28 days. Similarly, significant reductions were seen in the a- and b-wave amplitudes over the same period: a-waves were 72%, 69%, 58%, and 45% of their baseline at 1, 8, 14, and 28 days, and b-waves were 72% 66%, 66%, and 55% of their baseline at 1, 8, 14, and 28 days, respectively (*t*-test, p<0.001) (Figure 2, Figure 4B).

### Group IV

ERG traces from a representative mouse in group IV (n=13), recorded before laser treatment and 1, 7, 14, 28, 42, and 56 days after treatment, are shown in Figure 3. When laser-treated eyes are compared to contralateral untreated fellow eyes, mice in this group showed significant (*t*-test, p<0.001) reductions in pSTR of approximately 81%, 90%, 72%, 56%, 77%, and 74%, respectively, at 1, 7, 14, 28, 42, and 56 days. Similarly, there were significant reductions in the a- and b-wave amplitudes: Compared with the values recorded in their fellow eyes (*t*-test, p<0.001; Figure 4C) the a-wave amplitudes of the treated eyes were observed at 64%, 70%, 58%, 38%, 44%, and 54% at 1, 8, 14, 28, 42, and 56 days, respectively, while b-wave amplitudes were 54%, 64%, 60%, 47%, 49%, and 59%, respectively, for the same intervals.

### Time course of ocular hypertension-induced loss of retrograde-labeled retinal ganglion cells

**Figure 5:**
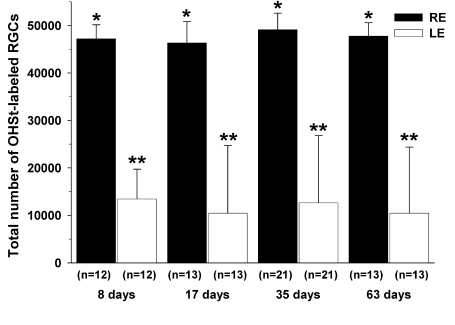
Populations of retinal ganglion cells labeled with 10% hydroxystilbamidine methanesulfonate at 8, 17, 35, and 63 days after lasering. Histograms represent the mean (±SD) total numbers of retinal ganglion cells (RGCs) labeled with 10% hydroxystilbamidine methanesulfonate (OHSt) in the left eye (LE) and right eye (RE) for groups I, II, III, and IV, analyzed 8, 17, 35 or 63 days, respectively, after laser treatment of the LE. To identify RGCs capable of retrograde axonal transport, OHSt was applied to both superior colliculi 1 week before sacrifice. Retinas were imaged with adjacent, nonoverlapping frames captured in a raster pattern. OHSt-labeled RGCs were counted in each frame using image analysis software. The average total numbers of OHSt-labeled RGCs in the LE were approximately one fourth of the total numbers of OHSt-labeled RGCs found in their untreated fellow retinas (RE) at all time points studied. The mean total numbers of OHSt-labeled RGCs in the RE for all four groups were comparable (*Kruskal–Wallis test; p=0.1391). Similarly, the mean total numbers of OHSt-labeled RGCs in the lasered retinas (LE) for these groups were also comparable (**Kruskal–Wallis test; p=0.2991). This suggests that the loss of retrograde axonal transport does not change beyond 8 days.

**Figure 6:**
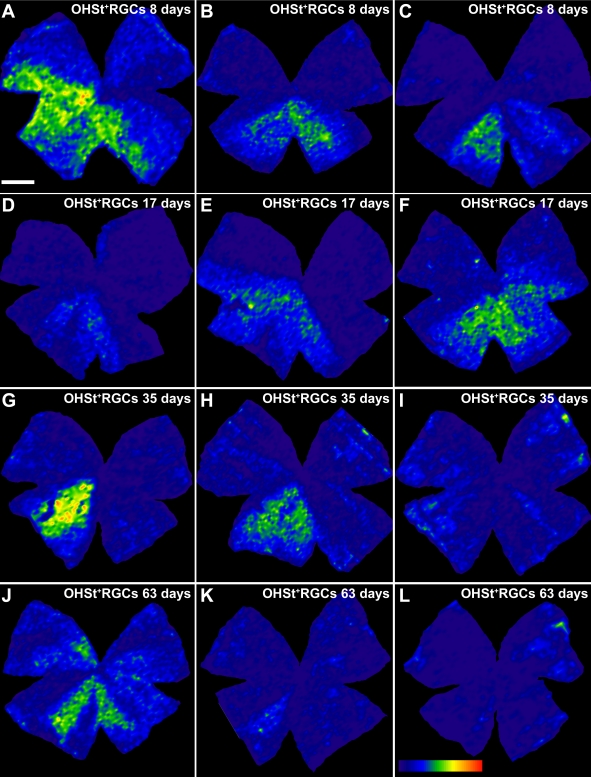
Isodensity maps of retinal ganglion cells labeled with 10% hydroxystilbamidine methanesulfonate in experimental retinas. The isodensity maps of representative experimental retinas from groups I (**A**-**C**), II (**D**-**F**), III (**G**-**I**), and IV (**J**-**L**) illustrate regions with different densities of retinal ganglion cells labeled with 10% hydroxystilbamidine methanesulfonate (OHSt^+^ RGCs). To identify RGCs capable of retrograde axonal transport, OHSt was applied to both superior colliculi one week before animal processing. Whole-mount reconstructions were prepared with the aid of a motorized stage on a photomicroscope with a high-resolution camera connected to an image analysis system (Image-Pro Plus, V5; Media Cybernetics, Silver Spring, MD). Retinal multi frame acquisitions were photographed in a raster scan pattern in which contiguous frames were captured with no gap or overlap. Isodensity maps were generated by assigning a color code to each of the 36 subdivisions of each individual frame according to its RGC density value within a 45-step color scale range, from 0 (dark blue) to 5,625 RGCs/mm^2^ or higher (red). For all retinas, the dorsal pole is orientated at 12 o’clock (scale bar=1 mm). Note that there are many regions with focal loss— that is, with almost no backlabeled RGCs — as well as regions with sparsely distributed RGCs. For example, the retina illustrated in E shows a region containing backlabeled RGCs restricted to a wedge between the 5 and 10 o’clock positions. The retina illustrated in A also shows backlabeled RGCs in higher numbers within a large wedge between 5 and 10 o’clock, but spared RGCs are also distributed throughout the rest of the retina in lighter densities, as reflected by the cooler than normal colors. In the four groups, approximately 91% of the retinas show areas with severe absence of RGCs. This absence is primarily (in 94% of the retinas) in the dorsal retina.

**Figure 7:**
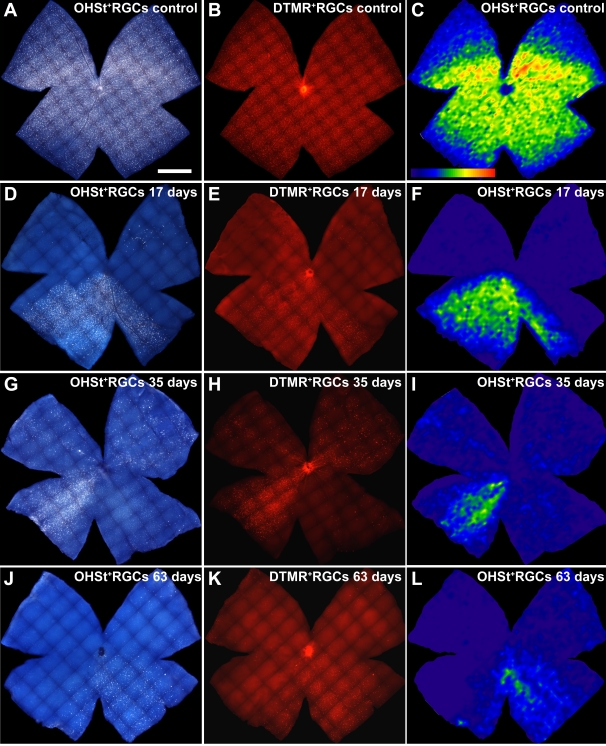
Isodensity maps of retinal ganglion cells labeled with dextran tetramethylrhodamine and 10% hydroxystilbamidine methanesulfonate in control and experimental retinas. Retinal whole-mounts of representative adult albino Swiss mice are shown at different survival intervals after lasering the left eye and treating with dextran tetramethylrhodamine (DTMR) and 10% hydroxystilbamidine methanesulfonate (OHSt). To identify retinal ganglion cells (RGCs) capable of retrograde axonal transport, OHSt was applied to both superior colliculi 1 week before animal sacrifice, and to identify RGCs with a competent axon at the level of the optic nerve (ON) head, DTMR was applied to the ocular stump of the intraorbitally divided ON 2 days before sacrifice. These retinal whole-mounts illustrate typical topological distributions of OHSt^+^ RGCs and DTMR^+^ RGCs throughout these retinas. Note the close correspondence of the areas lacking backlabeled RGCs with both tracers for each illustrated retina. For all retinas, the dorsal pole is orientated at the 12 o’clock position (scale bar=1 mm). **A**-**C**: Whole-mount of a representative control right retina double-labeled with OHSt (**A**) applied 1 week before dissection and DTMR (**B**) applied 5 days later. An isodensity map of OHSt-labeled RGCs was constructed by splitting each frame into 36 parts and estimating RGC densities (**C**). Intensely labeled RGCs are distributed throughout the retina, but higher densities are present in a region along the naso-temporal axis of the superior retina, resembling a visual streak [69]. **D**-**F**: Whole-mounts of a representative experimental left retina from Group II (17 days after lasering) show the lack of OHSt-labeled RGCs (**D**) in a large sector that comprises the superior and small sectors of the inferior retina. Stained with DTMR (**E**), the same retinal sector shows few to no DTMR-labeled RGCs. An OHSt-labeled RGC isodensity map (**F**) illustrates the areas lacking backlabeled RGCs in a sector that spans almost from the 4 o’clock position to half past 8 o’clock. **G**-**I**: These whole-mounts of representative experimental retinas from Group III (35 days after lasering) show important diminutions in the numbers of OHSt-labeled RGCs (**G**) in all quadrants of the retina, except for the inferior-nasal. When stained with DTMR (**H**), the same retinal quadrants show few to no DTMR-labeled RGCs. The OHSt-labeled RGC isodensity map (**I**) illustrates the areas that lack backlabeled RGCs. **J**-**L**: Whole-mounts of a representative experimental retina from Group IV (63 days after lasering) show a lack of OHSt-labeled RGCs (**J**) mainly within the nasal retina and the superior-temporal quadrant. Stained with DTMR (**K**), the same retinal quadrants showed few to no DTMR-labeled RGCs. The OHSt-labeled RGC isodensity map (**L**) also illustrates the areas with focal loss of RGCs, as well as a small sector of the inferior temporal quadrant that shows diffuse loss of backlabeled RGCs.

To determine the effects of elevated IOP on the retrograde axonal transport of OHSt, groups of mice were analyzed at increasing survival intervals of 8, 17, 35, and 63 days after lasering. Some variability was observed among animals belonging to the same group, in that individual retinas exhibited variable numbers of OHSt-labeled RGCs (Figure 5) and differed in corresponding areas of the retina without labeled RGCs (Figures 6 and Figure 7). Most of the lasered retinas analyzed at 8, 17, 35, and 63 days showed typical areas lacking RGCs labeled with OHSt that had been applied to both SCi one week before animal processing. The areas that lacked OHSt-labeled RGCs were comparable in the four groups of retinas (Figure 6).

Experimental retinas from the different groups showed mean total numbers of retrograde-labeled RGCs that were significantly less than in their untreated fellow retinas (Kruskal–Wallis test, p<0.0001). For group I, the treated eyes had approximately 28% of the OHSt-labeled RGCs in the untreated eyes (Mann–Whitney test, p<0.0001); for group II, 23% (Mann–Whitney test, p=0.0001); for group III, 26% (Mann–Whitney test, p=0.0000); and for group IV, 22% (Mann–Whitney test, p=0.0001) (Figure 5). The mean number of OHSt-labeled RGCs in the left (experimental) retinas of the groups sacrificed 8, 17, 35, and 63 days after lasering were 13,428±6,295 (n=12), 10,456±14,301 (n=13), 12,622±14,174 (n=21), and 10,451±13,949 (n=13), respectively, while the right (untreated) retinas presented mean total numbers of 47,223±2,936 (n=12), 46,321±4,492 (n=13), 49,101±3,489 (n=21) and 47,748±2,829 (n=13), respectively (Figure 5). The mean total numbers of OHSt-labeled RGCs in the contralateral retinas from the four groups were comparable (Kruskal–Wallis test, p=0.1391). Similarly, the mean total numbers of OHSt-labeled RGCs in the lasered retinas from the four groups were also comparable (Kruskal–Wallis test, p=0.2991), indicating that in this experimental paradigm, the lack of OHSt-labeled RGCs is present by 8 days after lasering and does not progress beyond that point (Figure 5**)**.

### Retinal distribution of retrograde-labeled retinal ganglion cells

Microscopic examination of the control fellow retinas showed the typical distribution of RGCs throughout the retina (Figure 7A–C), while the lasered retinas revealed large areas devoid of OHSt-labeled RGCs (Figure 6A–L and Figure 7D–L). This absence was seen in approximately 91% of the experimental retinas, in the form of a wedge-shaped space with an apical vertex toward the optic disc. Approximately 94% of the retinas showed these areas lacking RGCs backlabeled with OHSt in the upper retina. The pattern of OHSt-labeled RGC distribution within the retina was examined in detail by constructing an isodensity map for each retina (Figure 6 A–L). Representative examples that illustrate typical areas lacking OHSt-labeled RGCs, as well as their topological distribution, are shown in Figure 6 and Figure 7. When analyzed 8 (Figure 6A–C), 17 (Figure 6D–F and Figure 7D–F), 35 (Figure 6G–I and Figure 7G–I), and 63 (Figure 6J–L and Figure 7J–L) days after lasering, approximately 100%, 92%, 86%, and 92% of the retinas, respectively, showed areas lacking RGCs labeled with OHSt. These contour plot isodensity maps revealed that in addition to the retinal sectors that lacked backlabeled RGCs, there was also a diffuse absence of OHSt-labeled RGCs even within retinal areas where some labeled RGCs appeared (Figure 6 and Figure 7). Thus, the lack of OHSt-labeled RGCs within the OHT retinas was both focal and diffuse.

### Double labeling of retinal ganglion cells

Animals from all groups were backlabeled with OHSt applied to both SCi one week before sacrifice. In addition, in groups II (n=13), III (n=12), and IV (n=13), DTMR was applied to the ocular stump of the intraorbitally transected left optic nerve 5 days after OHSt application and 2 days before sacrifice. OHSt would identify RGCs that retained their retrograde axoplasmic transport capacities, while DTMR would identify RGCs with a competent axon at the level of the ON head. In general, there was a good correlation between the retinal areas lacking OHSt-labeled RGCs and the areas lacking DTMR-labeled RGCs (Figure 7A–L).

### Immunocytofluorescence studies

#### Brn3a immunostaining of the retina

**Figure 8:**
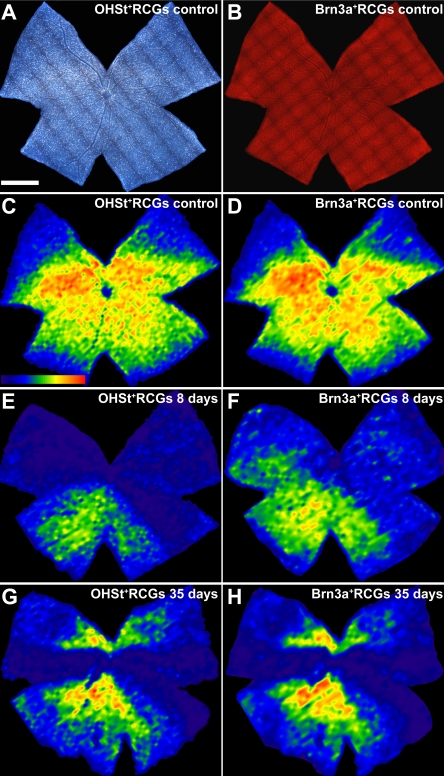
Absence of backlabeled retinal ganglion cells due to axonal transport impairment and cell loss. **A**-**H**: These representative examples show whole-mounts of a control retina (**A**-**D**) labeled with 10% hydroxystilbamidine methanesulfonate (OHSt) (**A**) and its isodensity map (**C**), as well as the same retina immunolabeled with Brn3a (**B**) to identify surviving RGCs and the corresponding isodensity map (**D**). To identify RGCs capable of retrograde axonal transport, OHSt was applied to both superior colliculi 1 week before sacrifice. To identify surviving RGCs, retinas were processed for Brn3a immunohistochemistry. Note the presence of intensely stained RGCs distributed throughout the entire control retina, with a typical high-density region along a naso-temporal streak in the superior retina, which is clearly shown in the isodensity maps of OHSt-labeled RGCs (**C**) and Brn3a-labeled RGCs (**D**). **E**-**H**: Representative examples of experimental retinas at 8 (**E**, **F**) and 35 (**G, H**) days after lasering, showing the isodensity maps of OHSt-labeled RGCs (**E**, **G**) and Brn3a-labeled RGCs (**F**, **H**). Both experimental retinas show fewer OHSt- and Brn3a-labeled RGCs than in the control retinas. Moreover, the population of Brn3a-labeled RGCs was greater than that of OHSt-labeled RGCs at 8 but not at 35 days, indicating compromised retrograde axonal transport within the first week after lasering and demonstrating that the lack of retrograde labeling in the retina is due not only to RGC degeneration but also to an impairment of the axoplasmic flow. The diminution in the numbers of Brn3a^+^RGCs observed between the control retina and retinas analyzed 8 and 35 days after lasering also documents the loss of RGCs. Isodensity maps were generated by assigning a color code to each of the subdivisions of each individual frame according to its RGC density value within a 45-step color scale range, from 0 (dark blue) to 5,625 RGCs/mm^2^ or higher (red). For all retinas, the dorsal pole is orientated at 12 o’clock (scale bar=1 mm).

To determine whether the absence of backlabeled RGCs represented actual RGC death or a functional impairment of axonal transport, retinal whole-mounts of mice analyzed 8 (n=12) or 35 (n=9) days after lasering were processed for Brn3a immunohistochemistry to identify and count surviving RGCs. These retinas had been labeled with OHSt applied to both SCi one week before sacrifice. Control retinas showed a typical distribution of OHSt-labeled RGCs throughout the retina (Figure 8A), and the retinal distribution of Brn3a-labeled RGCs (Figure 8B) paralleled that of OHSt-labeled RGCs (Figure 8 C,D), as recently described for rats [51]. The average total numbers of Brn3a-labeled RGCs in the right control retinas at 8 and 35 days were 49,375 (±2,822; n=12) and 50,577 (±2,036; n=9), respectively. The left LP retinas examined 8 and 35 days after lasering showed 24,343 (±5,739; n=12) and 10,219 (±8,887; n=9) Brn3a-labeled RGCs, respectively. Eight days after lasering, these numbers were greater than those obtained for the OHSt-labeled RGCs (13,428±6,295; n=12) (Mann–Whitney test, p=0.0007; Figure 8E–F), but 35 days after treatment the numbers were comparable (10,522±9,426; n=9; Mann–Whitney test, p=0.9397; Figure 8G–H). The progressive diminution of the total number of Brn3a-labeled RGCs in treated retinas analyzed 8 and 35 days after lasering (Mann–Whitney, p=0.0004) also documents the death of RGCs (Table 1).

**Table 1:** Total numbers of OHSt^+^RGcs or Brn3a^+^RGCs in control and injured retinas.

**RGC cells**	**8 days after laser photocoagulation**		**35 days after laser photocoagulation**	
**RE (n=12)**	**LE (n=12)**	**RE (n=9)**	**LE (n=9)**
Brn3a^+^RGCs	49,375±2,822†	24,343±5,739*#	50,577±2,036†	10,219±8,887**#
OHSt^+^RGCs	47,223±2,936†	13,428±6,295*	46,649±2,693†	10,522±9,426**

Number (mean±SD) of RGCs immunodetected for Brn3a or labeled with OHSt applied to both superior colliculi one week before animal sacrifice, in contralateral (RE) or injured (LE) retinas of mice examined 8 or 35 days after laser photocoagulation of the left eyes. *Mann–Whitney test, p=0.0007; **Mann–Whitney test, p=0.9397; # Mann–Whitney test, p=0.004; † Kruskal–Wallis test, p=0.0796.

#### RT97 immunostaining of the retina

**Figure 9:**
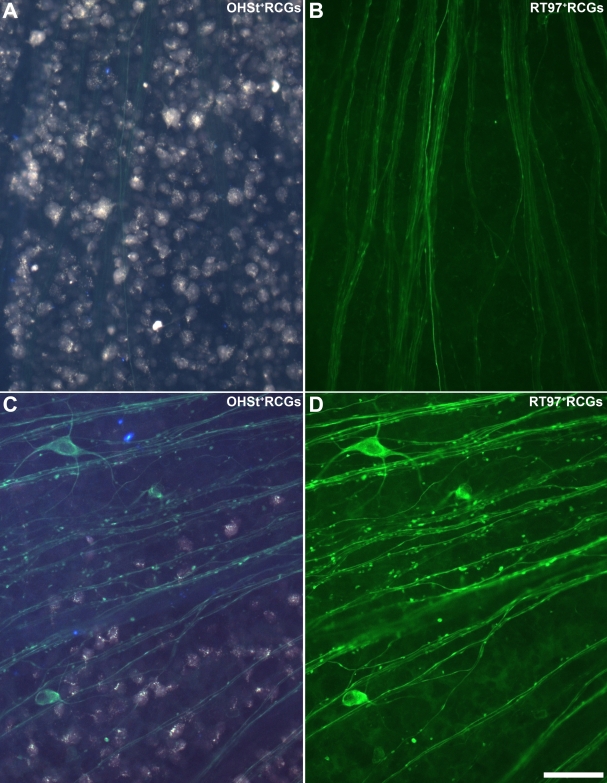
Ocular hypertension induces aberrant expression of phosphorylated neurofilament heavy chain. Fluorescence photomicrographs show representative middle regions of right eye (control; **A**, **B**) and left eye (lasered; **C**, **D**) mouse retinas, with 10% hydroxystilbamidine methanesulfonate (OHSt) applied to the superior colliculi for 1 week, that were processed for RT97 immunofluorescence 17 days after laser photocoagulation. Micrographs were taken under ultraviolet and fluorescein filters to examine OHSt or RT97 immunofluorescence, respectively. **A**, **B**: In the control (right-eye) retina, OHSt-labeled RGCs show typical distribution (**A**) and RT97 immunofluorescence is restricted to axonal bundles (**B**). **C**, **D**: In the lasered (left-eye) retina, the distribution of retinal ganglion cells (RGCs) retrograde-labeled with OHSt is restricted to the lower part of the picture (**C**), which under the fluorescein filter (**D**) shows a lack of RT97 staining within OHSt^+^ RGCs. Abnormal phosphorylated neurofilament heavy chain expression, which depicts several intensely RT97-stained RGCs and RT97 beaded axons, is concentrated in the region that lacks OHSt backlabeled RGCs. Scale bars for **A**-**D**=50 µm.

**Figure 10:**
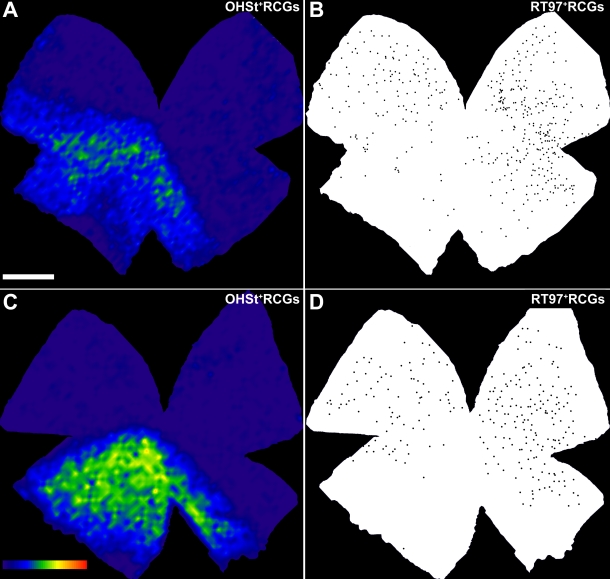
Different geographical distribution of RT97-labeled and OHSt-labeled retinal ganglion cells in retinas with ocular hypertension. These examples of representative left retinas in experimental animals 17 days after lasering the left eye show retinal ganglion cells (RGCs) labeled with 10% hydroxystilbamidine methanesulfonate (OHSt) and RT97, illustrating the near confinement of abnormal expression of RT97 to sectors of the retina that lack OHSt backlabeled RGCs; meanwhile, sectors containing OHSt^+^ RGCs present fewer RGCs with abnormal RT97 staining. To identify RGCs capable of retrograde axonal transport, OHSt was applied to both superior colliculi 1 week before sacrifice. Retinal whole-mounts were immunostained with RT97 antibodies to identify RT97^+^ RGCs. OHSt^+^ RGC isodensity maps were generated by assigning a color code to each of the 36 subdivisions of each individual frame according to its density value within a 45-step color scale, ranging from 0 (dark blue) to 5,625 RGCs/mm^2^ or higher (red). RT97^+^ RGCs are represented as dots over the outline of the retinal whole-mount. **A**, **B**: A whole-mount of a representative experimental left retina doubly labeled with OHSt (**A**) and RT97 (**B**) illustrates the typical topological distribution of 12,821 OHSt^+^ RGCs and 415 RT97^+^ RGCs throughout the retina in these experiments. Note the lack of correspondence between areas containing OHSt-labeled RGCs (**A**), which are restricted to a large wedge located between the 5 and 10 o’clock positions, and the RT97^+^ RGCs (**B**), which are distributed mainly in the opposite area of the retina. The dorsal pole of the retina is orientated at 12 o’clock. **C**, **D**: Another whole-mounted representative experimental left retina doubly labeled with OHSt (**C**) and RT97 (**D**) illustrates the topological distribution of 10,911 OHSt^+^ RGCs and 269 RT97^+^ RGCs throughout the retina in these experiments. Note the lack of correspondence between areas containing OHSt-labeled RGCs (**C**) which are restricted to a large wedge between 4:30 and 8:30 o’clock, and the RT97^+^ RGCs (**D**) found mainly in the opposite area of the retina. The dorsal pole of the retina is orientated at 12 o’clock (scale bar=1 mm).

Dissected retinas were stained with RT97, an antibody that recognizes phosphorylated neurofilament heavy chain (pNFH) and whose abnormal expression is an index of axonal injury [71,81-88]. This antibody has been recently studied after optic nerve axotomy, either by complete intraorbital optic nerve transection or crush [74] or after ocular hypertension in adult albino rats [26]. In the present study, fellow control retinas showed typical RT97 immunostaining confined to the most distal portion of the axons within the middle and central retina, where they group into bundles and converge at the optic disk. Rarely, an axon was stained to the retinal periphery, and RT97 staining was infrequently observed in the somas or dendrites of RGCs (Figure 9A,B). In contrast, the pattern of RT97 expression in LP retinas processed at 8, 17, 35, and 63 days after lasering showed typical signs of axotomy-induced neuronal degeneration [26,74] (Figure 9C,D). In general, abnormal RT97 staining consisted mainly of abnormal pNFH accumulations both in the intraretinal aspect of RCG axons (shaped like rosary beads and small varicosities) and also within the cell bodies and primary dendrites of some RGCs (Figure 9D). RT97 labeling within the cell soma and primary dendrites varied from a faint but clear staining to an intense labeling (Figure 9C,D). Abnormal expression of RT97 was confined mainly to the sectors of the retina that lacked backlabeled RGCs, while the areas showing OHSt-labeled RGCs presented fewer abnormalities in RT97 immunoreactivity, as recently described for other inherited mouse models of ocular hypertension [53-57] and in a rat model of ocular hypertension [26]. While there were no noticeable changes in RT97 expression eight days post-treatment when the retinas were examined at low magnification, numerous RGC bodies and proximal dendrites were positive for RT97 by 17 days after lasering (Figure 9C,D); still, it was rare to observe a OHSt^+^ RGC doubly labeled with RT97, or vice versa (Figure 9 and Figure 10). At 35 and 63 days, the abovementioned changes in the RT97 expression pattern within axons had evolved further, and there were fewer RT97^+^ RGCs.

In the five control retinas, the number of RT97^+^ RGCs in each was 0, 0, 12, 15, and 13, respectively, with a mean (±SD) of 8±7 (n=5). The 40 RT97^+^ RGCs found in control retinas were all faintly stained. In contrast, in the 5 experimental retinas, totals of 202, 415, 269, 165, and 291 RT97^+^ RGCs were counted in each individual retina, with a mean (±SD) of 268±96 (n=5). Out of a total of 1,342 RT97^+^ RGCs found in the experimental retinas, 27% were strongly labeled and 73% were faintly stained. The population of RGCs in Swiss mouse retinas has been recently shown to be of 49,493 RGCs [69]; thus, the population of RT97^+^ RGCs represented, in the best case, less than 1% of the total population of RGCs. Nevertheless, the number of RT97^+^ RGCs in the LP retinas showed a great increase when compared to control retinas.

In the retinas in which manual counts were performed, a comparison of the topological distribution showed that RT97^+^ RGCs were mainly distributed in regions that lacked OHSt-labeled RGCs. Two representative examples of this spatial analysis are provided in Figure 10.

In the wedge-shaped sectors devoid of OHSt^+^ RGCs and DTMR^+^ RGCs, normal-looking RT97 stained axonal bundles were observed converging toward the optic disk throughout the retina. By 63 days after LP, there was a diminution in the density of RT97-labeled axonal bundles in the central and peripheral retina, and RT97-stained somas in RGCs were infrequently observed.

## Discussion

The present study provides new information regarding the effects of elevated IOP on adult albino Swiss mice and extends our previous observations of the adult albino rat [26]. Lasering of the limbal and perilimbal veins resulted in an abrupt increase of IOP, already measurable at 24 h, that persisted until day 5, when IOP started to decrease gradually, reaching preoperative baseline values by one week post-treatment. Important diminutions in the numbers of backlabeled OHSt^+^ RGCs were already evident by 8 days after lasering and remained constant thereafter. Thus, the lack of OHSt^+^ RGCs occurred soon after lasering, did not progress between 8 and 63 days and in general approximately 25% of the original population of RGCs maintained their retrograde axonal transport at 8 days or greater survival periods examined. The geographical distribution of the backlabeled RGCs shows that elevated IOP induces — as has been shown in the albino rat [26] — both sectorial (in the form of wedge-shaped regions) and diffuse loss of RGCs. Our immunofluorescence studies also indicate that early after IOP elevation, retrograde axonal transport is impaired in a subpopulation of RGCs that are Brn3a^+^ and thus survive in the retina. RT97 immunostaining further shows that in the sectors of the retina lacking OHSt^+^ RGCs, some surviving RGCs show typical signs of crush-injury-like induced degeneration. Our electrophysiological analysis demonstrates that shortly after LP, there is an important reduction in the amplitude of the STR and the other two main components of the ERG, the a- and b-waves; these reductions are permanent throughout the period of study, indicating that elevation of IOP in albino mice results in damage not only to the RGC layer, but also to other retinal layers. All together, we found that lasering of the limbal tissues in albino mice results in profound alterations of retrograde axoplasmic transport that are followed by degeneration of RGCs, as well as important alterations of the innermost, inner nuclear, and outer nuclear layers of the retina.

### Elevation of intraocular pressure

Elevation of IOP, the primary risk factor for glaucoma, is also the most common approach to induce experimental models in which to study pathophysiology involved with glaucomatous optic neuropathy. Injection of hypertonic saline into the episcleral veins [89], cauterization of the episcleral veins [90], and laser photocoagulation of the trabecular meshwork [30] are some accepted approaches to induce ocular hypertension in animal models of glaucomatous damage. Much is known about inherited mouse models of glaucoma, the DBA/2J line of mice, and also about the experimentally induced models of glaucoma in pigmented mice [32,33,59], but little is known about the subject in albino mice.

The extent of IOP elevation varies with different experimental approaches and animal species [15,30]. Indeed, different lasering methods result in different IOP elevation profiles with variations in the time at which the peak IOP is reached and maintained, as well as differences in how long ocular hypertension persists [15,30,89]. The lasering methodology employed in the present study resulted in a rapid increase of IOP that was maintained for a short period of time. While this may be seen as a disadvantage when compared to a more chronic model of IOP elevation, our IOP profile produced a severe injury to the retina that resulted in several features — loss of RGCs and degeneration of the nerve fiber layer, for example — that are common in an inherited mouse model of ocular hypertension [54-57] commonly used to investigate mechanisms of cell death in glaucomatous optic neuropathy. Similar abrupt elevations of IOP have been observed for pigmented mice, in which the trabecular meshwork and the episcleral veins were photocoagulated with a laser [60]. The pigmented mice studied by Grozdanic and colleagues [12] showed persistent IOP increases for over 30 days, while in our study, the IOP elevation was restricted to the first week. Nevertheless, our results are somewhat comparable, in that they also show important alterations of the STR, a-waves, and b-waves of the ERG recordings, all of which imply severe retinal damage (see below).

### Absence of retrograde-labeled retinal ganglion cells

Evaluation of RGC survival was based on the use of specific markers and retrograde-transported tracers that allowed specific identification of RGCs over flattened mouse retina whole-mounts, along with the application of recently developed techniques to automatically count labeled RGCs and construct topological isodensity maps of their distribution within the retina. Quantitative analysis of the number of RGCs backlabeled with OHSt applied to both SCi showed substantial diminution of OHSt^+^ RGCs in lasered retinas when compared with their contralateral fellow retinas. Within each group examined at different survival intervals, there was some variability in the intensity of retinal damage, as judged by the size of areas lacking labeled RGCs and the total number of RGCs labeled with OHSt. At present, we have no clear explanation for the variability of retinal damage in the present work, but similar variations in the intensity of retinal damage have been reported in experimental [30] and inherited [55,56,59] models of ocular hypertension, including our recent study that used similar lasering methodology in adult albino rats [26]. There were no differences among the groups analyzed at different survival intervals; thus, it is conceivable that whatever damage was induced in these retinas occurred soon after injury.

The lack of retrograde-labeled cells in the experimental retinas took two primary forms: a focal absence, in wedge-shaped sectors with their vertex on the optic disc and their periphery toward the retinal periphery; and another, less conspicuous reduction that was diffuse and affected retinal areas containing some backlabeled RGCs. Focal loss occurred in wedges of different sizes within the retinal quadrants but more often in the dorsal retina, as has been previously observed in other ocular hypertension studies in rats [26,28,30,89] and mice [54,55,59,64]. We ignore the exact reason for the preferential location of retinal damage in the dorsal retina but suppose it may be related to the basic structure of the optic nerve. The spatial distribution of OHSt^+^ RGCs in the lasered retinas suggests a diffuse lack of backlabeled RGCs in addition to the focal loss of the cells. Indeed, the isodensity maps constructed on the basis of the OHSt^+^ RGCs provided a vivid demonstration of the presence of retinal areas with lower than normal RGC densities (Figure 6, Figure 7, and Figure 8). A similar pattern of RGC loss has been reported in aged DBA/2NNia mice [52] as well as in adult OHT rats [26].

### Functional impairment of retrograde axonal transport

The results for the experimental groups in which OHSt was used as a retrograde tracer to identify RGCs demonstrated large areas of the retina that lacked labeled RGCs. While the absence of backlabeled RGCs may reflect degeneration and death of RGCs, it might also be related to a functional impairment of axoplasmic flow, as has been previously shown for other retinal injuries in adult rats, such as transient ischemia [43] or ocular hypertension [26]. In these studies it was shown that a proportion of the RGC population that survives transient ischemia of the retina or ocular hypertension exhibits impairment of retrograde axonal transport [26,43]. To investigate whether the lack of OHSt-labeled RGCs was a reflection of axonal transport impairment, OHSt was applied to both SCi 1 wk before animal processing, and the retinas were processed for Brn3a immunohistofluorescence to identify and count surviving RGCs. There was a clear mismatch between the number of OHSt^+^ RGCs and the number of Brn3a^+^ RGCs, indicating that within the first days after lasering, retrograde axonal transport of OHSt from the SCi back to the RGC cell body is compromised. The number of surviving, functionally competent RGCs is much larger as judged by Brn3a expression, further demonstrating that not all surviving RGCs retain normal physiologic properties [26,43]. These experiments indicate that the lack of retrograde labeling in the retina, at the early time point of 8 days after lasering, is due not only to an actual degeneration and loss of RGCs but also to an impairment of the axoplasmic transport.

The absence of OHSt^+^ RGCs in the wedge-shaped sectors was paralleled by the lack of retrograde labeling with DTMR, suggesting that the scarcity of labeled RGCs is due also to a lack of passive diffusion along the RGC axon (Figure 7). It is possible that early after lasering, within 8 days of treatment, there is an actual impairment of active retrograde axonal transport of OHSt along the axon, while by 17 or more days after lasering, the lack of RGCs backlabeled with OHSt or DTMR responds to impairment of active retrograde axonal transport and of passive diffusion from the optic nerve head toward the cell body. This is consistent both with a mechanical obstruction altering passive diffusion and retrograde transport of DTMR and also with retrograde cell loss of unlabeled RGCs. Indeed, the observation that the number of Brn3a^+^ RGCs diminishes progressively from the control samples to retinas examined at 8 days and then retinas examined at 35 days provides evidence for the loss and death of RGCs. Thus, it seems that OHT induces not only a scarcity of labeled RGCs but also the death of RGCs.

### Axotomy-like injury post-ocular hypertension

The topologic distribution of RGCs in the form of triangular sectors suggests a lesion inflicted at a point where optic axons are topographically grouped together; this must occur at the level of the optic nerve head, where retinotopic arrangement is highest [91,92]. These wedge-shaped patterns of retinal damage are reminiscent of those observed in other types of inherited or acquired retinal degeneration, in which blood vessels ligate bundles of axons, thus producing an intense constriction that leads to axonal transport interruption, loss of retrograde tracing, and loss of RGCs [71,83-86]. The latter situation affects bundles of axons close to the optic disc, with effects that radiate out toward the periphery, but no anomalies are seen within unconstricted bundles of axons. The geographical distribution of areas without backlabeled RGCs in our present study sharply contrasts with the characteristic patchy cell loss observed after transient ischemia of the retina induced by selective ligature of the ophthalmic vessels [43,72,73].

It was interesting to find apparently normal neurofilament staining in sectors with labeled RGCs, but not in sectors lacking labeled RGCs (Figure 9C,D). In agreement with our double-labeling studies, which showed that a large number of surviving Brn3a^+^ RGCs had lost their capacity for retrograde axonal transport of OHSt, some RT97^+^ RGCs were found within the areas that lacked labeled RGCs, as were bundles of RT97^+^ retinal axons (Figure 9C,D and Figure 10). This suggests that retrograde degeneration and death of OHT-lesioned RGCs and their axons takes longer, as previously suggested [26,54,56]. The degenerative events in the ganglion cell layer following optic nerve injury vary depending on the type of injury and are somewhat slower when the injury is due to axonal compression than when it is due to axonal transection [74].

There was no indication of further diminution in the numbers of OHSt^+^ RGCs between 8 and 63 days after lasering. By 63 days, the retinas showed total numbers of OHSt^+^ RGCs that were not significantly different from those observed in the similar groups of animals processed at 8, 17, and 35 days after lasering, implying that perhaps the numbers of backlabeled RGCs do not decrease further with increasing survival intervals. Progressive loss of RGCs was observed when these neurons were identified with Brn3a immunofluorescence, which agrees with previous studies of RGC loss induced by an axotomy-like insult, such as ON transection [93], ON crush [74,94], or axon bundle ligation [71,83-86].

### Functional impairment of the retina (electroretinography)

Our previous studies on OHT-induced retinal degeneration in adult rats [26], together with the present studies, indicate that shortly after IOP elevation, severe pathology develops in the retina, affecting the RGC population. However, little is known about changes in retinal function in an OHT model induced by LP of episcleral and limbal veins in adult albino Swiss mice; thus, ERG were recorded in this study to examine functionally the progression of retinal damage in living animals induced by OHT over time in this experimental paradigm.

It is likely that IOP elevation results in permanent damage to the outer retinal layers, because in our experiments, both the a-wave and the b-wave amplitudes diminish soon after lasering and do not recover with time. Indeed, our results are consistent with other studies in rat OHT eyes showing severe alteration of the a- and b- waves shortly after OHT only when the peak of IOP is elevated [13,14,17,95,96]. Whether diminution in the amplitudes of the a- and b-waves reflects a functional impairment that precedes actual degeneration of the outer retinal layers remains to be studied further, but parallel studies conducted in our laboratory [97] suggest that the degeneration of the outer retinal layers is a protracted event following IOP elevation. In those studies, the morphology and general appearance of the outer retinal layers, as observed with several antibodies, do not appear altered shortly after lasering (two weeks post-treatment), but they become very distorted by 4 months after lasering. Our present results are also consistent both with other rat OHT eye studies that show cellular disorganization and thickness reduction of all retinal layers [12] and a severe thinning of the retinal structures [14] and also with reports in human advanced glaucoma that indicate altered scotopic ERG parameters [8] and thinning of the outer and inner nuclear layers with loss of photoreceptors [6].

Our results on the recorded a-waves and b-waves illustrate damage to the outer and middle retinal layers, but they provide no information about the innermost retina. Recording STR, the lowest detectable response in scotopic conditions, appears to be a fine and sensitive functional test to detect damage to the innermost layer of the retina, specifically involving the RGC population [13,98]. In both pigmented [13,37] and albino [38] rats, the STR diminishes significantly shortly after optic nerve transection, a diminution that correlates with the pattern of axotomy-induced RGC loss and persists for long periods of time without recovery, indicating that the integrity of the RGC population is needed to evoke a full STR in these rodents. Our measurement of ERG responses using a series of flashes with varying light intensity allowed us to evaluate the functional changes longitudinally; the results showed that the STR was severely affected shortly after the rise in IOP, and this alteration was maintained throughout the longest survival interval examined, 8 weeks, thus providing additional functional evidence of the loss of RGCs in this model. This is consistent with previous OHT studies in adult albino rats [26], as well as with our present anatomic data indicating degeneration of the RGC population. Moreover, a significant reduction in STR has been associated with chronic [14,17,60,99,100] and acute [95,101] OHT in adult rats, mice [96], and monkey OHT models and human glaucoma patients [8,98].

Thus, taken together our present data shows that elevation of IOP in albino mice results in important diminutions of the STR, a-wave, and b-wave amplitudes, which are generated by the innermost, outer and inner nuclear layers of the retina, respectively. A similar effect has been observed in rat and monkey models of glaucoma [14,98]. To our knowledge, we report for the first time significant decreases in STR, a-wave, and b-wave ERG scotopic responses in adult albino Swiss mice with elevated IOP. Correspondence in physiologic measurements in this mouse OHT model indicates the value of noninvasive ERG measurements to document retinal pathophysiology in rodent glaucoma models.

### Concluding remarks

Our data indicate that OHT in adult albino Swiss mice induces several severe degenerative events in the retina. The first event observed in our experiments is the loss of the capacity for active retrograde axonal transport from target territories in the brain back to cell soma in the retina. This is seen within a week of retinal lasering. Shortly after, axonal transport appears blocked for bundles of neighboring axons, in such a way that even a passively diffusing retrograde tracer does not reach the somas of the surviving RGCs in the retina; this appears between 8 and 17 days after lasering and is maintained for the period of study. At the early time point (8 days), however, there are still larger numbers of RGCs distributed throughout the retina, as identified by the expression of the RGC-specific marker Brn3a. Simultaneously, the nerve fiber layer of the retina shows signs of retrograde degeneration of axons and RGC bodies that are compatible with an axotomy-like injury, and the chronology of the degenerative events speaks in favor of a crush-like injury to bundles of axons in the ON head. Up to 63 days post-treatment, the retinas exhibit a small proportion (approximately 22%) of the original RGC population that maintains their capacity both for active retrograde transport of OHSt from their target territories in the brain back to the retina and for passive retrograde diffusion of DTMR from the ON head toward the cell soma in the retina. These events are paralleled by an important functional impairment that consists of reductions in the amplitudes of the a- and b-waves and the STR wave to approximately half of their basal levels. These reductions, which are observed as early as 24 h after laser treatment, are maintained for as long as 8 weeks, suggesting permanent damage to the innermost, inner nuclear and outer nuclear layers of the retina.

The mechanism by which the outer, inner and innermost retinal layers are compromised in the present model is not fully understood, but it may result from different types of insults. It has been largely known that increased IOP may mimic the effect of an axotomy-like injury to RGC axons somewhere near the optic nerve head [26,102-104], where ON axons are highly arranged retinotopically [91,92]. Additionally, the inner retinal blood supply may also be compromised by ON head compression; thus, a vascular compromise or mechanical compression of retinal vessels cannot be disregarded completely [105-107]. Finally, astrocytes have also been implicated in the structural remodeling that occurs in the optic nerve head of glautomatous eyes, thus glial activation may also be an important contributor to glaucomatous optic neuropathy [56,108].
